# Bacterial Communities in Three Parts of Intestinal Tracts of Carpenter Bees (*Xylocopa tenuiscapa*)

**DOI:** 10.3390/insects11080497

**Published:** 2020-08-03

**Authors:** Phakamas Subta, Phongsathon Yodsuwan, Rujipas Yongsawas, Ammarin In-on, Natapot Warrit, Somsak Panha, Kitiphong Khongphinitbunjong, Panuwan Chantawannakul, Korrawat Attasopa, Terd Disayathanoowat

**Affiliations:** 1 Department of Biology, Faculty of Science, Chiang Mai University, Chiang Mai 50200, Thailand; toy_subpata@hotmail.com (P.S.); karn.yodsuwan@gmail.com (P.Y.); r.yongsawas@gmail.com (R.Y.); Panuwan@gmail.com (P.C.); 2 Bioinformatics & Systems Biology Program, King Mongkut’s University of Technology Thonburi (Bang Khun Thian Campus), Bang Khun Thian, Bangkok 10150, Thailand; ammarin.ammarinin@mail.kmutt.ac.th; 3 Center of Excellence in Entomology, Department of Biology, Faculty of Science, Chulalongkorn University, Bangkok 10330, Thailand; natapot.w@chula.ac.th; 4 Department of Biology, Faculty of Science, Chulalongkorn University, Bangkok 10330, Thailand; somsak.pan@chula.ac.th; 5 School of Science, Mae Fah Luang University, Chiang Rai 57100, Thailand; khongphinit@gmail.com; 6 Department of Entomology and Plant Pathology, Faculty of Agriculture, Chiang Mai University, Chiang Mai 50200, Thailand; k.attasopa@gmail.com; 7 Research Center in Bioresources for Agriculture, Industry and Medicine, Chiang Mai University, Chiang Mai 50200, Thailand; 8 Research Center of Microbial Diversity and Sustainable Utilization, Chiang Mai University, Chiang Mai 50200, Thailand

**Keywords:** carpenter bees, intestine, microbiota, next-generation sequencing

## Abstract

This study investigated different bacterial communities in three intestinal parts (foregut, midgut and hindgut) of *Xylocopa* *tenuiscapa* to understand the roles of gut bacteria. Our phylogenetic analysis revealed that *X. tenuiscapa* is closely related to *Xylocopa latipes*. The 16S rRNA gene in the genomic DNA samples from the gut was examined by illumina (Solexa) and a total of 998 operational taxonomic unit (OTUs) clusters were found. Taxonomic classification identified 16 bacterial phyla and unclassified bacteria. The dominant bacteria taxa in the three parts of *X. tenuiscapa* gut were Proteobacteria, Firmicutes, Bacteroidetes and Actinobacteria. In the foregut, Lactobacillales and Enterobacteriaceae were predominantly found. The population in the midgut was similar to that in the foregut, with the addition of *Gilliamella*, which was also abundant. The most dominant bacteria identified in the hindgut were similar to those in the midgut and Lactobacillales, Enterobacteriaceae, *Gilliamella*, Bifidobacteriaceae and Flavobacteriaceae appeared in abundance. Moreover, our results suggest that a community structure of bacteria in different parts of *X. tenuiscapa*’s gut may be an important indicator of carpenter bees’ health. This functional study of bacterial communities revealed significant differences among the three intestinal parts and is the first report of the gut bacteria structure in solitary bees.

## 1. Introduction

Carpenter bees are insect pollinators that play an important role in the sexual reproduction of plants in tropical ecosystems. They are more efficient than honey bees because of the larger body size that allows for more pollens to adhere. Previous research reported that carpenter bee *Xylocopa olivacea* can increase the pollination efficiency of *Phaseolus vulgaris* [1]. Moreover, *X. varipuncta* is an important pollen carrier for mangroves in Setiu Wetland, Terengganu [2]. Hongjamrassilp et al. (2014) analyzed the pollen composition on the body of *X. nasalis* in Ratcha Buri province, Thailand, and found that the pollens were mostly native to the region of Southeast Asia, suggesting that *X. nasalis* is highly valuable for crop pollination [3]. Furthermore, stingless bees, *Apis* honey bees and *Xylocopa* carpenter bees are major pollinators in highly populated, tropical cities such as Bangkok [4].

The study of the gut microbiota of bees is an area that has received attention because the gut contains microorganisms that affect bees’ health. Microorganisms such as lactic acid bacteria (LAB) and actinobacteria support bees in digesting and providing nutrients, aiding against pathogens and strengthening the immune system [5]. For instance, LAB isolates from bees can inhibit *Paenibacillus larvae* that cause American foulbrood in bees [6]. They are considered probiotic and produce antibiotics against bacteria whose biofilm prevents grams and carbohydrate degradation [7,8,9].

The intestinal tract of a bee has three sections (foregut, midgut and hindgut). The foregut spans from the mouth cavity to the proventriculus. The midgut is adjacent to the foregut and is responsible for the digestion and absorption of food. The hindgut contains ileum and rectum. The bacteria community in an intestinal tract of *Apis mellifera* has been studied by qPCR and fluorescence in situ hybridization (FISH) and it was found that there were fewer bacteria in the foregut and midgut than in the hindgut. Particularly in the ileum, *Snodgrassella alvi, Gilliamella apicola, Lactobacillus* Firm-4 and *Lactobacillus* Firm-5 were dominant while *Lactobacillus* Firm-4 and *Lactobacillus* Firm-5 mostly populated the rectum [10,11]. However, most research studies on bacteria communities in bees focused on highly social bees in the genus *Apis* and in bumble bees and none have studied carpenter bees [12,13,14].

This study aimed to examine the bacterial community in intestinal tracts of carpenter bees. We utilized next generation sequencing (NGS) to sequence the 16s rRNA gene of bacteria in carpenter bees’ guts and identified bacterial communities in each section. Classification of bacteria was performed and phylogenetic trees were constructed. The functions related to the bacterial community cluster were predicted and we found that the structure of bacteria in the gut may contribute to the health of bees.

## 2. Materials and Methods

### 2.1. Collection of Carpenter Bees

**Figure 1:**
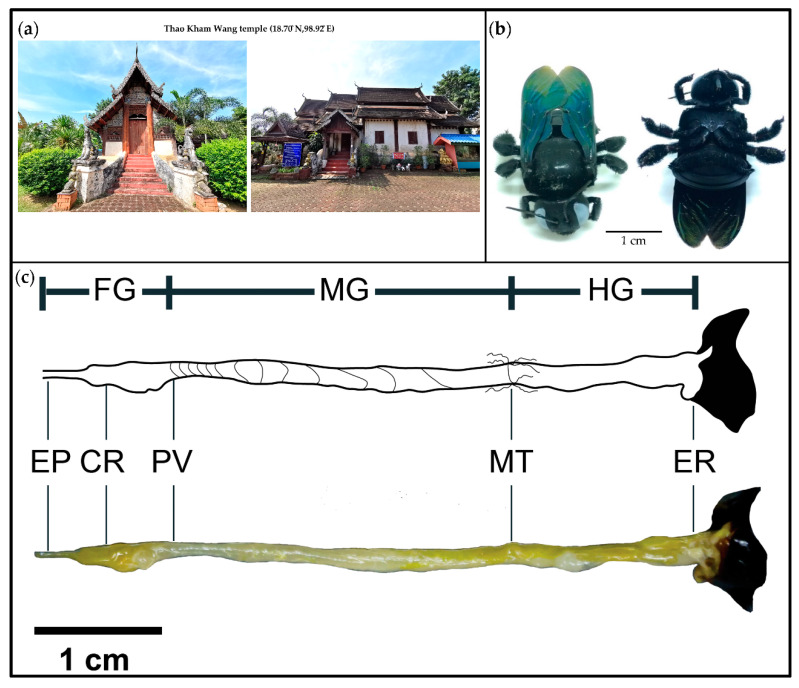
The old wooden church in Thao Kham Wang temple where carpenter bees live (**a**), carpenter bees that were collected from the church (**b**) and three sections of a carpenter bee’s intestinal tract, FG—foregut, MG—midgut and HG—hindgut, with details of EP—esophagus, CR—crop, PV—proventriculus, MT—malpighian tubule and ER—end of rectum (**c**).

A total of six pollinating carpenter bee samples were collected around the Thao Kham Wang temple area (18.70° N, 98.92° E) (Figure 1a) in October 2018 in Chiang Mai Province by an aerial net. Samples were collected in a box. Carpenter bee specimens were morphologically identified as *X. tenuiscapa* using identification keys of Hurd and Moure (1963) [15]. Individual carpenter bee samples (Figure 1b) were surface sterilized [16] with 70% NaClO for 1 min, 95% ethanol for 1 min, 70% ethanol for 30 s and sterile water for 3 min. Three parts of each gut were dissected under a stereomicroscope (Figure 1c) [17]. The foregut spanned the esophagus and crop to the proventriculus. The midgut contained the ventriculus. The hindgut extended from the pylorus and small intestine to the end of the rectum (before the sting apparatus) [10,18]. Then, individual parts of the intestinal tract (foregut, midgut and hindgut) (Figure 1c) were separated from the body and put in 500 µl PBS buffer in a 1.5 mL centrifuge tube to culture LAB and for NGS.

### 2.2. Classification of Carpenter Bee Samples

Genomic DNA of samples were extracted by DNaeasy Blood & Tissue Kit (QIAGEN, Germantown, MD, USA) and amplified the COI gene by forward primer LepF (5′-ATTCAACC AATCATAAAGATAT-3′) and reverse primer LioR (5′-CCAAAAAATCAAATAAATGTTG-3′) [19]. The PCR condition was as follows: denaturation at 94 °C for 30 s, annealing at 55 °C for 30 s, extension at 72 °C for 45 s and final extension at 72 °C for 7 min. DNA samples were sequenced by sanger sequencing. A phylogenetic tree was constructed using the unweighted pairs group method with arithmetic mean (UPGMA) on MEGA 7 [20].

### 2.3. DNA Extraction and NGS of Gut Sections

DNA extracts from gut sections were used as templates to amplify the 16S rRNA gene by primers S-D-BACsT-1494-A-S-20 (GTCGTAACAAGGTAGCCGTA) and L-D-BACT-0035-A-A-15 (CAAGGC ATTCACCGT) [21]. The PCR condition was as follows: initial denaturation at 95 °C for 5 min, denaturation at 95 °C for 30 s, annealing at 53 °C for 2 min, extension at 72 °C for 2 min and final extension at 72 °C at 10 min. PCR products were stored in −20 °C.

#### 2.3.1. Next Generation Sequencing

NGS of each intestinal section (foregut, midgut and hindgut) was performed for 16S amplicon (V3-V4 regions) using illumina (solexa) by Macrogen (Macrogen Inc., Seoul, Korea). The sample preparation protocol for 2 × 300 bp paired-end reads was applied, with a number of read average ranging from 150k to 260k bp.

#### 2.3.2. Data Analysis

A library construction was performed and edited by Mothur V 1.41.1 [22]. Briefly, we used “make.contigs” to merge paired-end sequences and cut poor sequences using “screen.seqs” with average quality control of read >q30; non 16S sequences were filtered out and chimeric sequences were removed by “chimera.uchime”. We clustered sequences into operational taxonomic units (OTUs) using “dist.seqs” and “cluster” 97% identity sequence to operational taxonomic unit (OTU) clustering by the Greengenes 13.8 database. OTUs were subjected to BLAST against the NCBI database nucleotide collection for alpha diversity. The Simpson diversity index, Shannon diversity index and principle coordinated analysis (PCoA) were performed by Past V.3 and non-metric multidimension scaling (NMDS) were performed by R program V.3.6.0. Lastly, multivariate analysis of variance, PCoA test and one-way permutational multivariate analysis of variance (PERMANOVA) were performed by Past V.3 and NMDS test two-way PERMANOVA was performed by R program V.3.6.0.

The representative sequences (Table S1) were obtained from the Mothur results using “get.oturep” with cut off = 0.03. Then, a representative sequence of each taxon was aligned against the HoloBee database V.2016.1 [23], and the four top-hit species were added to the pre-multiple sequence alignment (MSA) sequence list. The pre-MSA sequence list contained representative sequences which were used as the input of webPRANK [24] and for MSA analysis to build the phylogenetic tree. The tree was exported as a newick file. Python 3.6 base was used for re-visualizing the phylogenetic tree, as shown in Figure 6.

#### 2.3.3. Prediction and Analysis of Functions of the Bacterial Microbiota

Phylogenetic Investigation of Communities by Reconstruction of Unobserved States (PICRUSt) 1.0.0 (http://picrust.github.io/picrust) was used to predict the predictive functional profiling of microbial communities using 16S rRNA marker gene sequences. The functions were analyzed based on clusters of orthologous groups (COGs) [25].

#### 2.3.4. NGS Data Accession Number

The results from this study have been submitted to the NCBI database with the SRA accession PRJNA525318.

## 3. Results

### 3.1. Classification of Carpenter Bee Samples

**Figure 2:**
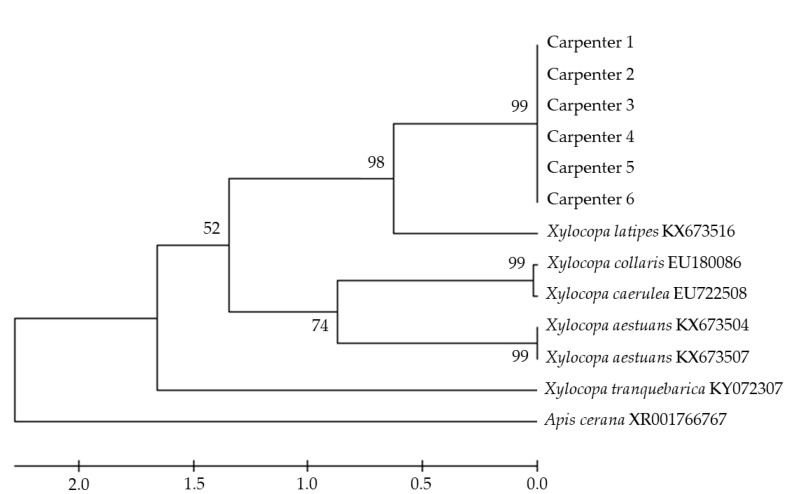
Phylogenetic tree of *Xylocopa* spp. COI gene sequences and closely related reference sequences were constructed by unweighted pairs group method with arithmetic mean (UPGMA). Bootstrap values (based on 1000 resamplings) higher than 50 were indicated at the nodes.

Six sequences of genomic DNA from carpenter bee samples were aligned with six sequences of *Xylocopa* spp. genomic DNA identified as closely related references in MEGA 7. We used the genomic DNA sequence of *Apis cerana* as an out group. Phylogenetic analysis with 1000 bootstrap resamplings indicated that the six samples were in the same group, with 98% sequence identity to *X. latipes* (Figure 2).

### 3.2. Sequencing Results

**Figure 3:**
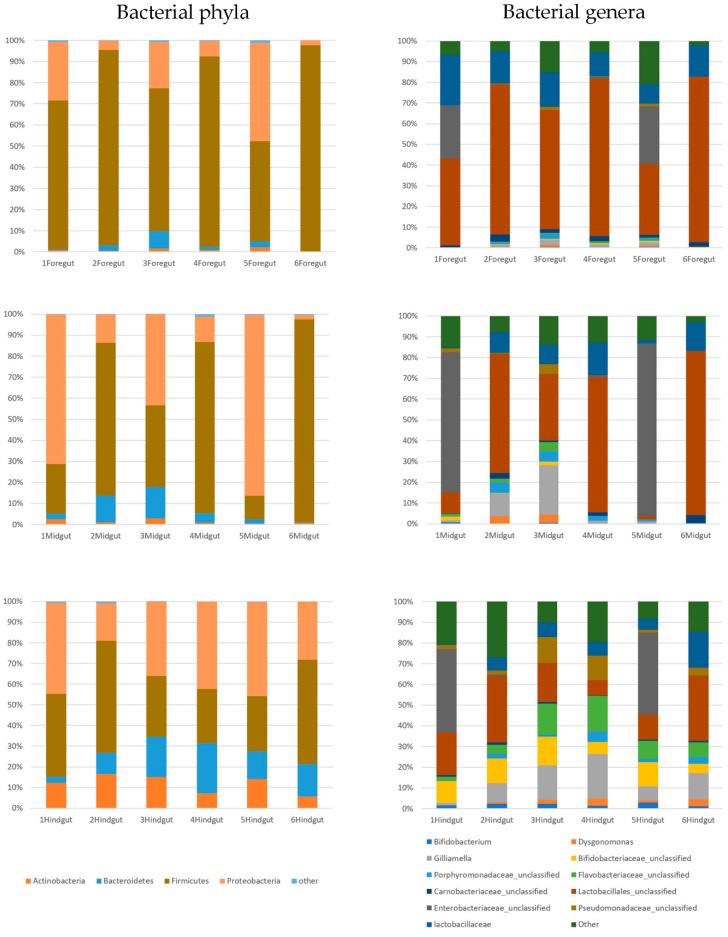
Bacterial communities in the foregut, midgut and hindgut. Other operation taxonomic unit (OTU) bacteria in samples with less than 2% prevalence are included as “other” category.

We investigated bacteria in the gut of six *Xylocopa* (six sets of foreguts, midguts and hindguts). All 18 samples were sequenced by the illumina MiSeq platform using the 16S rRNA gene. A total of 998 OTUs clusters were found. Taxonomic classification identified 16 bacterial phyla and unclassified bacteria in the carpenter bee gut. The majority of sequences were those of the Proteobacteria, Firmicutes, Bacteroidetes and Actinobacteria (Figure 3). The results showed that the dominant bacteria identified in the foregut were Firmicutes (77.17%), most of which belonged to the order Lactobacillales (48.44%) and family Carnobacteriaceae (2.08%), followed by Proteobacteria (18.20%), most of which were in the family Enterobacteriaceae (9.03%), and 0.28% were in the genera *Gilliamella*. The most dominant bacteria identified in the midgut were similar to those in foregut. The prominent phyla were Firmicutes (53.7%), most of which belonged to order Lactobacillales (40.93%), followed by Proteobacteria (37.47%), most of which belonged to family Enterobacteriaceae (25.05%), and 6.37% were from genera *Gilliamella*. Moreover, *Bacteroides* (6.23%) were also more abundant in the midgut, most of which were of genera *Porphyromonas* (2.27%). The most dominant bacteria identified in the hindgut were similar to those in the midgut but somewhat different to those in the foregut. The prominent phyla were Firmicutes (37.75%), with order Lactobacillales (20.47%) and family Lactobacillaceae (7.13%) as the most abundant, followed by Proteobacteria (28.37%), most of which belonged to family Enterobacteriaceae (13.43%), family Pseudomonadaceae (5.53%) and 11.27% of genera *Gilliamella*. In addition, the members of *Bacteroides* (14.32%) and Actinobacteria (1.40%) were more abundant in the hindgut, and these mostly were of family Flavobacteriaceae (6.16%) and *Porphyromonas* (2.23%), and 1.80% of genus *Dysgonomonas* belonged to phylum Bacteriodetes and 9.7% of family *Bifidobacteriaceae* belonged to phylum Actinobacteria (Figure 3 and Table S1).

**Figure 4:**
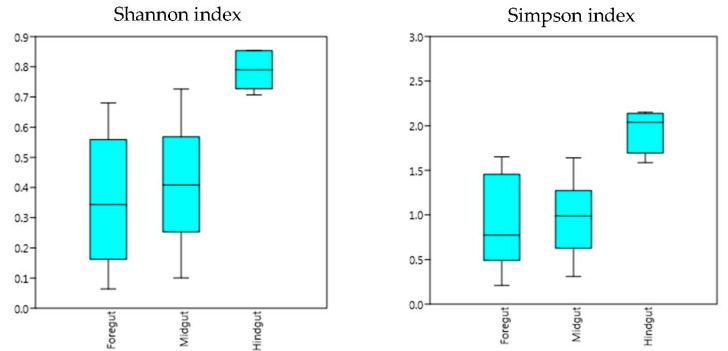
Shannon and Simpson diversity of microbial communities in each intestinal section (foregut, midgut, hindgut), based on plain numbers of OTUs.

**Figure 5:**
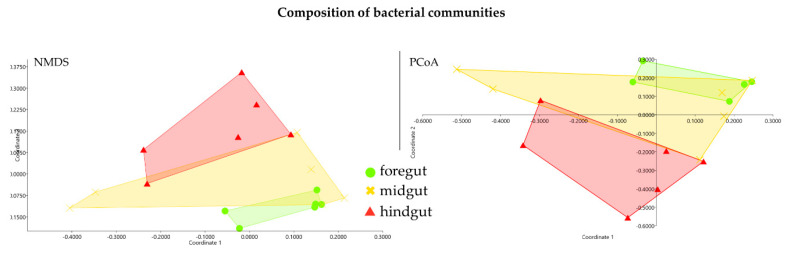
Ordination plots showing composition of bacterial communities in the intestinal tracts. Non-metric multi-dimensional scaling (NMDS) and principle coordinated analysis (PCoA) based on Bray–Curtis dissimilarity matrix were used for all samples. Calculations of dissimilarity were based on relative abundances of OTUs. Sample points were shaped by gut composition.

The results from the Simpson and Shannon diversity index showed that the microbial community in the hindgut was significantly different from that in the foregut and midgut (*p* < 0.01) (Figure 4). However, the results from NMDS and PCoA were different from those of the Simpson and Shannon diversity index, which showed that the bacteria diversity identified in the foregut was significantly different from that in the hindgut. Moreover, the bacteria diversity in the midgut was not significantly different from either the foregut or midgut (Figure 5). Future studies with a more diverse population and higher sample number may be able to confirm the findings of this study.

**Figure 6:**
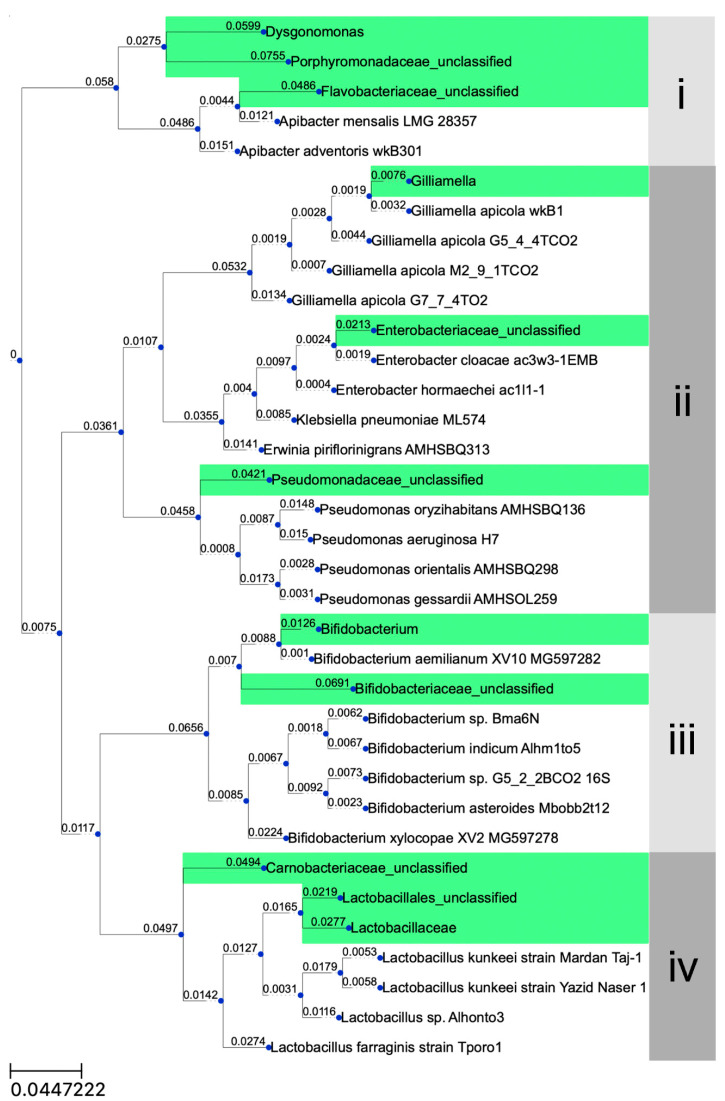
Multiple sequence alignment was performed using webPRANK, the phylogeny-aware algorithm which was suitable for aligning the gapped sequences. The length and the number of each tree branch represent the distance, as the scale bar shows the distance length of each branch.

Phylogenetic affiliation of nine core bee gut bacteria within *Xylocopa*-associated OTUs is shown in the green highlight in Figure 6. Phylogenetic analyses included additional core bee gut bacteria from HoloBee database. The sequences revealed the placement of the OTUs in four clusters (Figure 6): (i) *Dysgonomonas*, Porphyromonadaceae_unclassified and Flavobacteriaceae_unclassified in this study had no similarity with the same taxon from core bacteria in bees (80% similarity) but Flavobacteriaceae_unclassified were closely related to two *Apibacter* spp.; (ii) *Gilliamella*, Enterobacteriaceae_unclassified and Pseudomonadaceae_unclassified OTUs from *Xylocopa* were closely related to OTUs from honey bees; (iii) two representative *Bifidobacterium* from *Xylocopa* were closely related to *Bifidobacterium aemilianum* isolated from *Xylocopa* in the previous study [26]; (iv) *Lactobacillus* and Lactobacillales_unclassified were related to *Lactobacillus kunkeei* but were not closely related OTUs from honey bees with Carnobacteriaceae_unclassified.

**Figure 7:**
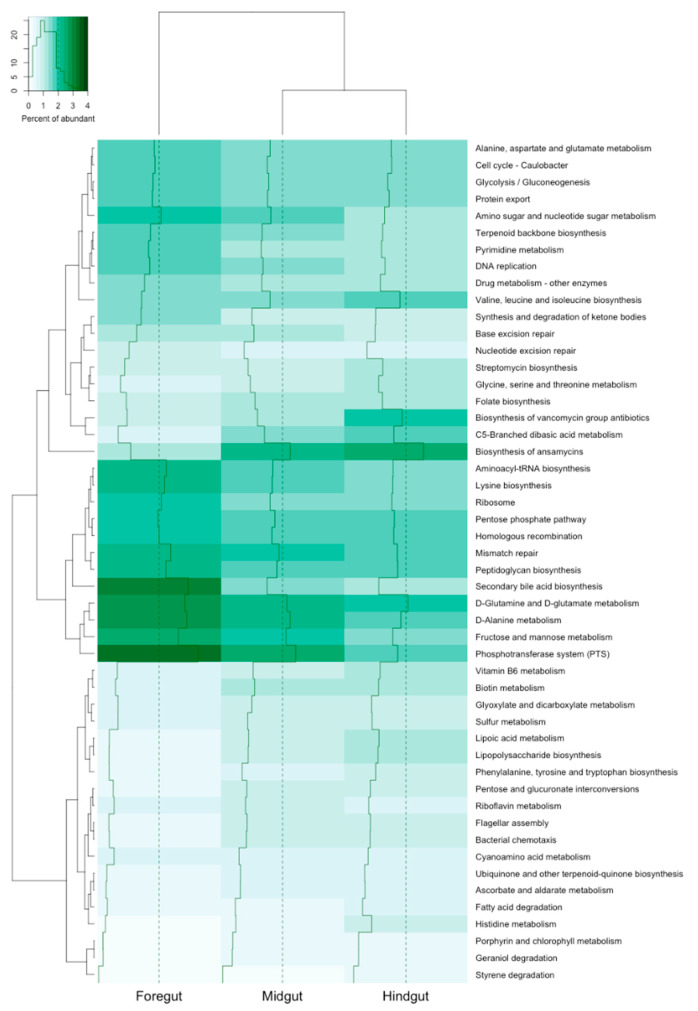
Hierarchical clustering graphics of the gene functions distance of bacterial communities (left) among foregut, midgut and hindgut (top) of *Xylocopa tenuiscapa* intestinal tracts by cluster analysis. The percent of functional gene abundance is shown on the top left with distribution of counts (*y*-axis). List of functional genes is shown on the right.

**Figure 8:**
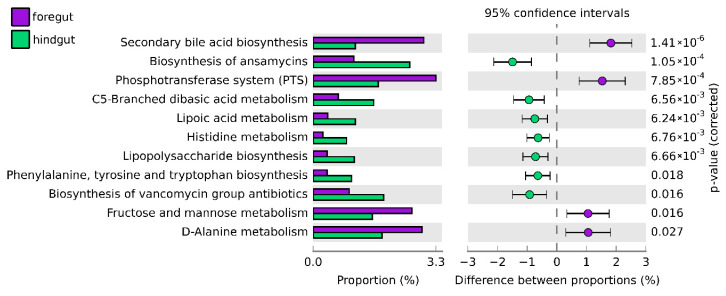
The significant differences in relative abundance of predicted gene proportion between the foregut and hindgut of *Xylocopa tenuiscapa*.

The heat map revealed that the most abundant functional gene groups were secondary bile acid biosynthesis and the phosphotransferase system (PTS) from foregut bacterial communities (Figure 7). There were significant differences across all of the functional genes between the foregut and hindgut of *Xylocopa* intestinal tracts (*p* < 0.05; Figure 8). A group of biosynthesis genes was prevalent in the foregut to hindgut, including those involved in secondary bile acid, phosphotransferase system, fructose and mannose metabolism and D-alanine metabolism. For the hindgut tract, functions that were related to protein metabolism and biosynthesis were significantly higher than those from the foregut, including dibasic acid, lipoic acid metabolism and histidine metabolism and lipopolysaccharide, phenylalanine, tyrosine, tryptophan and vancomycin group antibiotic biosynthesis (Figure 8).

## 4. Discussion

This study demonstrated that there were differences in bacterial diversity in different parts of the gut of carpenter bees in Thailand. The results showed that dominant phyla of gut microbiota in the carpenter bee were Proteobacteria, Firmicutes, Bacteroidetes and Actinobacteria, which were consistent with the previous study [27]. Although the gut microbiota mostly consisted of Proteobacteria and Firmicutes in bees, moths and termites, Bacteroidetes and Actinobacteria were also found in high abundance [12,28,29].

The bacterial diversity in the carpenter bee’s gut was found to be lower than that in termites and beetles but higher than that in Lepidoptera [28,29,30,31]. However, in comparison to other honey bees analyzed using culture-independent methods, the diversity in carpenter bees was similar [13,17,32,33]. Moreover, the phylogenetic tree revealed that OTUs from this study were closely related to with OTUs from other bees (Figure 6). Our results showed that the dominant bacteria from the three gut regions were from genera *Gilliamella*, family Lactobacillaceae (most of which were order Lactobacillales), family Carnobacteriaceae, family Enterobacteriaceae and family Bifidobacteriaceae. Lactobacillaceae and Bifidobacteriaceae were families of LAB, including genus *Lactobacillus* and genus *Bifidobacterium* [34]. *Lactobacillus* and *Bifidobacterium*, two of the most important genera within LAB, were commonly found as commensals and considered probiotics in humans and animals [35]. *Lactobacillus* and *Bifidobacterium* were recently identified in the stomachs of honey bees (Figure 6), *A. mellifera* [6,13,36,37], and functioned in carbohydrate breakdown and fermentation [37,38,39,40]. Other important roles include defending hosts from parasites and pathogens, as they have been shown to inhibit other microorganisms on culture plates, including honey bee pathogenic bacteria *Paenibacillus larvae* [9,38,41,42]. Therefore, *Lactobacillus* and *Bifidobacterium* have the potential to enhance the immune response and may be important for honey bees’ health [43].

*Gillamella* is another important group of bacteria that was most abundant in the honey bee’s gut [13,22,31,36] and the *Gillamella* OTU in this study was closely related to previous OTUs of *G. apicola* from other bees [44] (Figure 6). Although *Gillamella* are not classified as LAB, they provide similar functions. Studies have reported that *Gillamella apicola* were fermentative bacteria in the honey bee’s gut and supported hosts in the utilization, fermentation and uptake of some essential sugars that were indigestible by bees [45,46,47]. Similar to *Lactobacillus* and *Bifidobacterium*, *Gillamella* have been shown to protect hosts from pathogens. In honey bees, *Gillamella* produce a biofilm on an ileum wall [10,45] and may provide a barrier to prevent the attachment or entry of gut pathogens. Thus, *Lactobacillus*, *Bifidobacterium* and *Gillamella* may play similar roles in carpenter bees’ nutrient uptake, digestion and protection against parasites and pathogens by enhancing the immune system. Another dominant family of bacteria in the gut of the carpenter bee was Enterobacteriaceae. Most bacteria in this family have various metabolic abilities, including fermenting carbohydrates and contributing to nitrogen intake by nitrogen-fixing activity [48]. Some bacteria in the family Enterobacteriaceae were identified in honey bees, but their functions in bees have not yet been investigated [13]. In addition, the family Carnobacteriaceae was also identified in diamondback moths, *Plutella xylostella*, but their functions are still unknown [49].

We found that bacteria genus *Porphyromonas* and family Flavobacteriaceae were dominant in the midgut and hindgut. Members of this genus are normally identified from greengenes databases. *Porphyromonas gingivalis* has been shown to increase the metabolic inflammation risk and autoimmune disorders in mice [50]. Members of the family Flavobacteriaceae were identified in melon flies, *Zeugodacus cucurbitae* [51] as well as in the guts of honey bees [17]. The Flavobacteriaceae_unclassified OTU from this study (Figure 6) was related to *Apibacter mensalis*, which was isolated from *Bombus lapidarius* [52], and *Apibacter adventoris* isolated from honey bees and bumble bees [53]. However, *Bifidobacterium*, *Dysgonomonas* and family Pseudomonadaceae were found to be predominant only in the hindgut. *Dysgonomonas* had been identified in many insects’ guts [51,54,55]. It is involved in lignocellulose degradation. Members of the family Pseudomonadaceae were identified in cowpea beetles, *Callosobruchus maculatus* [55] and honey bees’ guts [13].

The functional analysis of the 16S rRNA gene based on the COG database suggested that functional genes involved in the phosphotransferase system (PTS) and fructose and mannose metabolism were dominant in the foregut compared to the midgut tract. It is possible that the high abundance could provide an early stage of protein and sugar breakdown, especially pollen and fructose, which are major components in nectar, to maximize the growth and reproduction of bacteria in other parts of the gut. These functions correlated with the abundance of the phylum Firmicutes, which plays major roles in biomass degradation in insects [56].

The comparison of gut bacteria diversity in the foregut, midgut and hindgut showed that the diversity of bacteria in the foregut and hindgut was significantly different but those in the midgut were not significantly different to either the foregut or hindgut. This is possibly due to the midgut being connected to the foregut and hindgut, thus allowing some bacteria to pass through. Moreover, the similarity in the bacteria diversity may be attributed to the comparable functions of the midgut to both the foregut and hindgut [57]. The significant difference in bacteria population between the foregut and the hindgut may be due to the length of the midgut, which may hinder the movement of bacteria from the foregut to the hindgut. Furthermore, the functions of the foregut are different from those of the hindgut, and therefore different colonies of bacteria would be needed for their distinct functions.

## 5. Conclusions

We investigated the bacterial communities in the carpenter bee’s gut. Our results showed that the core bacteria phyla that were found most abundantly were Firmicutes, Proteobacteria, Bacteroidetes and Actinobacteria, which were similar to those in honey bees and bumble bees. Most bacteria that were found in carpenter bees’ guts may be involved in nutrient absorption, protection against parasites and pathogen modulation. The bacterial communities in each part of the gut may be linked to the functions of each part and indicative of the health of bees. This study is the first report on the gut bacterial diversity of carpenter bees found in Asia.

## Supplementary Materials

The following are available online at https://www.mdpi.com/2075-4450/11/8/497/s1, Table S1: The operation taxonomic unit (OUT) proportion table of 12 dominant bacteria from 3 sections of *Xylocopa tenuiscapa* intestines.
