# Supplementary material for: Bacterial Communities in Three Parts of Intestinal Tracts of Carpenter Bees (Xylocopa tenuiscapa)
Source: Insects. 2020 Aug 3;11(8):497. doi: 10.3390/insects11080497 (PMC7469164; doi:10.3390/insects11080497)
Supplement: Supplementary file 1 [file insects-11-00497-s001.pdf]

Gut secsions	Sample numbers	Bifidobacterium	Dysgonomonas	Gilliamella	Bifidobacteriaceae _unclassified	Porphyromonadaceae_unclassified	Flavobacteriaceae _unclassified	Carnobacteriaceae _unclassified	Lactobacillales_unclassified	Enterobacteriaceae_unclassified	Pseudomonadaceae_unclassified	lactobacillaceae family	Other
Foregut	1	0	0	0	0	0.17	0	1.08	41.99	25.64	0	24.4	6.72
	2	0.22	0.11	1.35	0.11	0.79	0.45	3.48	72.5	0.11	0.22	15.6	5.05
	3	0.51	1.02	2.55	0.34	2.38	0.51	1.7	57.56	0	1.53	16.81	15.11
	4	0.16	0.16	1.26	0.63	0.16	0.79	2.52	76.1	0.79	0.47	11.48	5.5
	5	0.4	0.4	1.86	0.66	1.06	0.53	1.46	34.22	27.72	1.33	9.42	20.95
	6	0	0	0.36	0.18	0	0	2.19	79.96	0	0	15.3	2
Midgut	1	0.64	0.18	0.92	1.74	0.18	1.19	0.18	10.27	67.37	1.74	0.27	15.31
	2	0.09	3.6	10.98	0.28	5.02	1.8	2.65	57.39	0.09	0.28	9.94	7.86
	3	0.5	4	23.6	1.75	4.87	4.62	0.75	32.08	0	4.62	9.36	13.86
	4	0	0	1.51	0	2.27	0	1.76	64.99	1.01	0	15.62	12.85
	5	0	0	1.18	0	0.84	0	0	1.85	82.86	0	1.68	11.6
	6	0	0	0	0	0.35	0	3.85	78.98	0	0	13.66	3.15
Hindgut	1	1.66	0	11.13	10.54	0.09	11	0.7	20.82	40.16	1.92	0.52	2
	2	2.32	0.84	19.15	11.98	2.26	12.19	1.29	31.96	0.77	1.93	6.19	1.13
	3	2.43	1.87	16.54	13.83	1.03	14.95	0.84	18.88	0	12.43	7.1	10.09
	4	1.43	3.49	21.47	15.86	4.99	17.19	0.16	7.37	0.08	11.81	6.42	4
	5	2.9	1.26	16.67	11.54	1.73	8.71	0.47	12.24	39.48	1.41	1.18	1
	6	1.22	3.32	12.66	14.54	3.32	6.9	0.87	31.53	0	3.76	17.38	1

Supplementary table 1. The operation Taxonomic Unit (OUT) proportion table of 12 dominant bacteria from 3 section of *Xylocopa tenuiscapa* intestines.
